# Proteomic characterization of bovine granulosa cells in dominant and subordinate follicles

**DOI:** 10.1186/s41065-019-0097-5

**Published:** 2019-06-25

**Authors:** Qingling Hao, Zhiwei Zhu, Dongmei Xu, Wenzhong Liu, Lihua Lyu, Pengfei Li

**Affiliations:** 1 College of Life Science, Shanxi Agricultural University, Taigu, 030801 Shanxi China; 2 College of Animal Science and Technology, Shanxi Agricultural University, Taigu, 030801 Shanxi China

**Keywords:** Bovine, Follicle, Label-free, Proteomic analysis

## Abstract

**Background:**

Characterization of molecular factors regulating ovarian follicular development is critical to understanding its functional mechanism of controlling the estrous cycle, determining oocyte competency, and regulating ovulation. In previous studies, we performed next-gene sequencing to investigate the differentially expressed transcripts of bovine follicular granulosa cells (GCs) at the dominant follicle (DF) and subordinate follicle (SF) stages during the first follicular wave. This study aims to investigate the proteomic characterization of GCs of DF and SF in the bovine estrous cycle.

**Results:**

In total, 3409 proteins were identified from 30,321 peptides obtained from liquid chromatograph-mass spectrometer analysis. Two hundred fifty-nine of these proteins were found to be expressed differently in DF and SF. Out of 259, a total of 26 proteins were upregulated (fold change≥2) and 233 proteins were downregulated (fold change≤0.5) in DF. Gene Ontology (GO) analysis of proteome data revealed the biological process, cellular component and molecular function of expressed proteins in DF and SF, while the Kyoto Encyclopedia of Genes and Genomes (KEGG) pathway analysis showed important signaling pathways associated with follicular development such as the PI3K-Akt, estrogen, and insulin signaling pathways. Immunoblotting results of OGN, ROR2, and HSPB1 confirmed the accuracy of the data. Bioinformatics analysis showed that 13 proteins may be linked to follicular development.

**Conclusions:**

Findings from this study will provide useful information for exploring follicular development and function.

**Electronic supplementary material:**

The online version of this article (10.1186/s41065-019-0097-5) contains supplementary material, which is available to authorized users.

## Introduction

Bovine ovaries contain many follicles. Of these, only the DF has potential for ovulation. DF is, therefore, a critical source of biomarkers of ovarian follicular development and function. Bovine ovarian follicles grow in a wave-like pattern with typically 2 or 3 follicular waves per estrous cycle. Most of the follicles finally atresia during the process of follicular development, with one larger follicle ovulating and hence achieving ‘dominance’[1, 2]. In general, only the DF eventually ovulates during the last wave of follicular growth. In mammals, ovarian follicle development and atresia are complex, involving cell replication, specialization, differentiation and death. Previous studies reported a variety of hormones and growth factors that are closely associated with follicular development. GCs secrete hormones during follicle growth, particularly steroid hormones close to ovulation, such as progesterone and estradiol, which regulate critical phases of the reproductive cycle, including oocyte growth and maturation [3]. Estrogen plays a beneficial role in follicular development by reducing atresia and promoting GCs proliferation [4–6]. Conversely, androgens have been considered inhibiting to folliculogenesis [7, 8]. Follicle stimulating hormone (FSH), an endocrine factor, is critical to ovarian follicles growth because it regulates GCs proliferation and prevents apoptosis and estradiol production [9]. Gonadotropins and luteinizing hormones both regulate ovarian follicle growth and development [10]. Paracrine and autocrine factors such as insulin-like growth factor family, transforming growth factor β family, and fibroblast growth factor family modulate the response of follicle cells to gonadotropin signals [11]. Follistatin is involved in follicle cell proliferation, oocyte maturation, steroidogenesis and corpus luteum function [12, 13]. Cocaine and amphetamine-regulated transcript (CART), a pituitary gonadotropin, is involved in regulation of GCs estradiol production [14].

Despite our detailed knowledge of the hormones and growth factors that control ovarian follicular development (summarized above), the mechanism that regulates the selection of one developing follicle to gain dominance and continue to grow in each follicular wave remains unknown. Complete processing of DF maturation is complex, and multiple proteins are involved in this process to regulate functional differentiation of the follicles, so we used a proteomics approach to characterize the proteomic profile of bovine follicles and identify potential regulatory proteins and signaling pathways involved in follicular development and function. The present study can be used as a reference for further study of regulation mechanisms of follicular development in monotocous species.

## Materials and methods

### Animal care

All animal procedures were implemented in strict accordance with the principles outlined in “Guide for the care and use of Laboratory Animals” by the National Institute of Health.

### Collection of GCs

The ovaries were collected from three Holstein dairy cows in October 2017, and follicular growth was observed and recorded using daily ultrasonography. After 5–7 days of estrus, ovaries were removed from cows when the largest follicle appeared and the growth rate was significantly higher than the growth rates of other follicles. The largest follicle and second largest follicle were collected. Each of the follicles was dissected into two halves, the GCs were removed by gentle scraping of the follicle wall with microsurgery forceps, and detached cells were obtained by washing two times with DPBS, a mixture of DPBS and GCs was centrifuged in Eppendorf tubes, the supernatant was discarded and stored at − 80 °C for further analysis.

### Extraction and digestion of total proteins

GCs were lysed by ultrasonication and homogenization in a cold extraction buffer. Protein concentrations were determined using the Bradford Coomassie® Brilliant Blue G-250 method (BCA) according to manufacturer instructions and using bovine serum albumin (BSA) as a standard. 50 μg of protein from each sample were denatured, followed by the addition of 10 mM dithiothreitol and incubation for 1 h at 56 °C. Proteins were then alkylated with 55 mM iodoacetamide in the dark for 40 min at room temperature and were further subjected to digestion with 1 μg trypsin for 16–17 h at 37 °C.

### Mass spectrometry (MS) analysis

The content of each peptide was determined and quantified by Capillary High Performance Liquid Chromatography (Eksigent 425, AB SCIEX) coupled with label-free mass spectrometry (LC-MS/MS). (A) H_2_O + 0.1% formic acid and (B) acetonitrile + 0.1% formic acid, were parts of the mobile phase. Desalting of the samples was performed online using a reversed-phase C18 trapping column (0.1 mm internal diameter, 20 mm length, 3 μm particle size; Waters). The peptides were then separated using a nano-column (0.75 mm internal diameter, 150 mm length, 5 μm particle size; Waters) at 0.3 μL/min. Peptides were eluted from the column using the following gradient: 5–80% B for 110 min, 80–5% B for 0.1 min, maintained at 5% for 120 min and then returned to the initial conditions. A mass spectrometer (Q-Exactive; Thermo Scientific, USA) was connected to the liquid chromatography apparatus to detect the eluted peptides. The separated peptide fragments were identified using a mass spectrometer operated in positive ion mode with electrospray ionization and collision-induced dissociation (CID). Full-scan MS spectra (350–1750 m/z) was acquired at a resolution of 70,000 with an automatic gain control (AGC) target value of 3e6 by electrospray ionization. The full-scan maximum injection time was 20 ms (millisecond), and the dynamic exclusion was set to 25.0 s. CID spectra were acquired at a resolution of 17,500 with an AGC target value of 2e5 and a maximum injection time of 80 ms. The isolation window was set to 2.0 m/z.

### Data analysis

Raw data were imported into the Expressionists software (Proteome Discover 2.0) for processing, after which the quantification was performed based on the peak intensities of the report ions of the only unique peptides in the MS/MS Spectra. MS/MS spectra were searched against the Uniprot-COW FASTA database with the following mascot parameters: peptide mass tolerance for ±15 ppm and fragment mass tolerance for 20 mmu. Trypsin was used as the protein-cleaving enzyme, and the two missed cleavages were accepted. Carbamidomethylation of cysteine was designated as a fixed modification, and oxidation of methionine, acetylation on protein N-term were selected as variable modifications. The peptide confidence was high, peptide length was set to > 4, and peptide false discovery rate (FDR) was set to ≤0.01.

### Bioinformatics analysis

GO analysis was used to classify the functions of the differentially expressed proteins (fold change ≥2), which could be categorized into three main categories: biological process, cellular component and molecular function. The most important biochemical metabolic pathways and signal transduction pathways were identified by KEGG pathway analysis. The GO analysis and KEGG pathway analysis were realized using the Protein Analysis Through Evolutionary Relationships (PANTHER) database (http://www.pantherdb.org).

### Immunoblotting assay

To validate the global proteomics methodology, three proteins were selected to be measured by immunoblot analysis. The whole protein was extracted from bovine follicles for each group (DF and SF) using a protein extraction kit (Beyotime, China) according to manufacturer instruction. Protein concentrations were measured by the BCA method using BSA as the standard. Extracts were denatured at 97 °C for 5 min. Equal amounts (25 μg/lane) of protein from each sample were separated using 12% sodium dodecyl sulfate polyacrylamide gel electrophoresis (SDS-PAGE) and electro-blotted onto nitrocellulose (NC) membranes. Membranes were then incubated with primary antibodies (polyclonal antibody) at the following dilutions: anti-OGN (HA1136, 1:1000, Huaan, China), anti-ROR2 (HA1137, 1:500, Huaan, China), anti-HSPB1 (HA1138, 1:500, Huaan, China), anti-β-actin (1:1000, CWBIO, China). Primary antibodies were diluted in TBST and incubated overnight at 4 °C. After washing in TBS with 0.1% Tween-20, the membranes were incubated with horseradish peroxidase conjugated secondary antibody (1:10,000, CWBIO, China) for 2 h at room temperature. After washing, the membranes were detected using the eECL Western Blot kit (CWBIO, China) and exposed to film. The intensity of signals for each protein was quantified using Image-Pro Plus Software, version 6.0 (Media Cybernetics, USA) and normalized to values obtained from β-actin. All experiments were performed in triplicates.

## Results

### Top 40 highly expressed proteins in DF and SF

The top 40 highly expressed proteins in GCs of bovine DF and SF are shown below (Table 1). Many of them are known to be critical for follicular growth and development, including VIM, GSTA3, ATP5B, HSPA8, GAPDH, ATP5A1, EEF1A1, CYP11A1, KRT8, etc.

**Table 1:** Top 40 highly expressed proteins in GCs of bovine DF and SF

Gene symbol	Protein name	DF mean	SF mean
ALB	BOVISerum albumin	3.00498E+ 11	1.66381E+ 11
VIM	Vimentin	1.33143E+ 11	1.47405E+ 11
ACTG1	Actin, cytoplasmic 2	49,261,000,000	59,596,333,333
HIST1H2BI	Histone H2B	46,500,666,667	46,801,000,000
GSTA3	BOVIGlutathione S-transferase	37,623,900,000	36,703,633,333
SERPINH1	SerpiH1	18,890,000,000	31,953,666,667
HIST1H2AC	Histone H2A	22,751,333,333	27,639,333,333
HBB	Emoglobisubunit beta	40,723,333,333	25,048,666,667
HSPA5	78 kDa glucose-regulated protein	9,895,900,000	19,931,533,333
ATP5B	ATP synthase subunit beta, mitochondrial	17,337,333,333	19,779,666,667
HBA	Emoglobisubunit alpha	38,072,666,667	18,919,466,667
HSP90B1	Endoplasmin	10,696,733,333	18,827,100,000
TUBB4B	Tubulibeta-4B chain	8,715,066,667	18,603,000,000
PDIA3	Proteidisulfide-isomerase	15,069,666,667	17,569,933,333
–	Tubulialpha-1B chain	7,831,366,667	16,726,333,333
3 SV	Histone H4	17,524,333,333	16,164,000,000
HSPA8	Heat shock cognate 71 kDa protein	7,069,933,333	12,705,833,333
GAPDH	Lyceraldehyde-3-phosphate dehydrogenase	6,952,100,000	12,486,566,667
HSPB1	Heat shock proteibeta-1	5,331,333,333	12,345,666,667
CSE1L	Exportin-2	10,924,600,000	12,341,533,333
ENO1	Alpha-enolase	7,287,233,333	12,038,133,333
MDH2	Malate dehydrogenase, mitochondrial	11,867,200,000	11,311,366,667
P4HB	Proteidisulfide-isomerase	7,845,966,667	11,253,566,667
ATP5A1	ATP synthase subunit alpha	8,206,900,000	11,035,500,000
1 SV	Uncharacterized protein	18,568,900,000	10,807,066,667
C3	Omplement C3	13,413,333,333	10,005,600,000
CALR	CALR protein	6,769,066,667	9,786,833,333
EEF1A1	Elongatiofactor 1-alpha 1	4,380,900,000	9,154,200,000
HSPD1	60 kDa heat shock protein, mitochondrial	6,450,766,667	8,975,633,333
CKAP4	Uncharacterized protein	6,837,433,333	8,510,966,667
CYP11A1	Cholesterol side-chaicleavage enzyme, mitochondrial	3,307,000,000	8,183,440,000
ANXA6	AnnexiA6	4,137,566,667	7,952,200,000
PDIA6	PDIA6 protei(Fragment)	5,622,233,333	7,834,433,333
GANAB	Uncharacterized protein	7,330,300,000	7,730,266,667
KRT8	Keratin, type II cytoskeletal 8	692,036,666.7	7,465,050,000
HNRNPA2B1	Heterogeneous nuclear ribonucleoproteins A2/B1	4,744,266,667	7,143,933,333
PPIB	Tidyl-prolyl cis-trans isomerase B	5,321,533,333	6,926,133,333
PPIA	Tidyl-prolyl cis-trans isomerase A	2,491,466,667	6,321,800,000
OAT	Ornithine aminotransferase, mitochondrial	2,239,963,333	6,249,833,333

### Identification of differentially expressed proteins

Based on a fold change greater than 2 with *p* < 0.05, 259 differentially expressed proteins were identified in GCs between DF and SF, including 26 upregulated proteins (fold change≥2) and 233 downregulated proteins (fold change≤0.5) in DF (Additional file 1).

### GO analysis

**Fig. 1:**
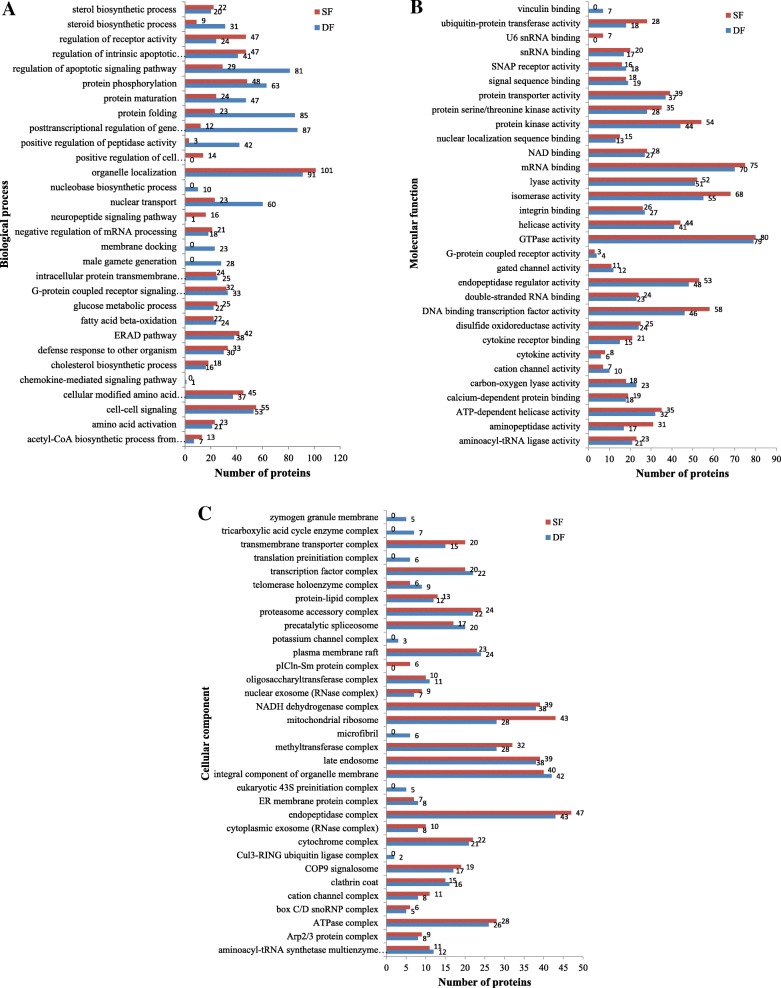
The expressed protein GO annotation. Classification of the expressed proteins in GCs of DF and SF in cattle based on the GO annotation, respectively. **a**: biological processes, **b**: molecular function, **c**: cellular components

A total of 3409 proteins were identified from the 30,321 peptide sequences obtained from LC-MS/MS (FDR ≤ 0.01). Two thousand eight hundred ninety-five proteins and three thousand one hundred two proteins were identified in DF and SF, respectively. The expressed proteins in DF and SF were categorized under 3 major GO classifications: biological process, cellular component and molecular function. Among the biological processes, many biological processes were associated with follicular development, such as regulation of the apoptotic signaling pathway, the steroid biosynthetic process, the sterol biosynthetic process, the male gamete generation process, the G-protein coupled receptor signaling pathway, and the cell-cell signaling process (Fig. 1a). Regarding most molecular functions, the number of proteins in DF was similar to the number of proteins in SF. For example, 79 proteins in DF and 80 proteins in SF were involved in GTPase activity, 51 proteins in DF and 52 proteins in SF were involved in lyase activity, 37 proteins in DF and 39 proteins in SF that possessed the function of protein transporter activity were detected, etc. (Fig. 1b). Among cellular components, most of the proteins were assigned to the endopeptidase complex, an integral component of the organelle membrane, late endosome, mitochondrial ribosome and NADH dehydrogenase complex (Fig. 1c).

### Biological processes classifications of differentially expressed proteins

**Fig. 2:**
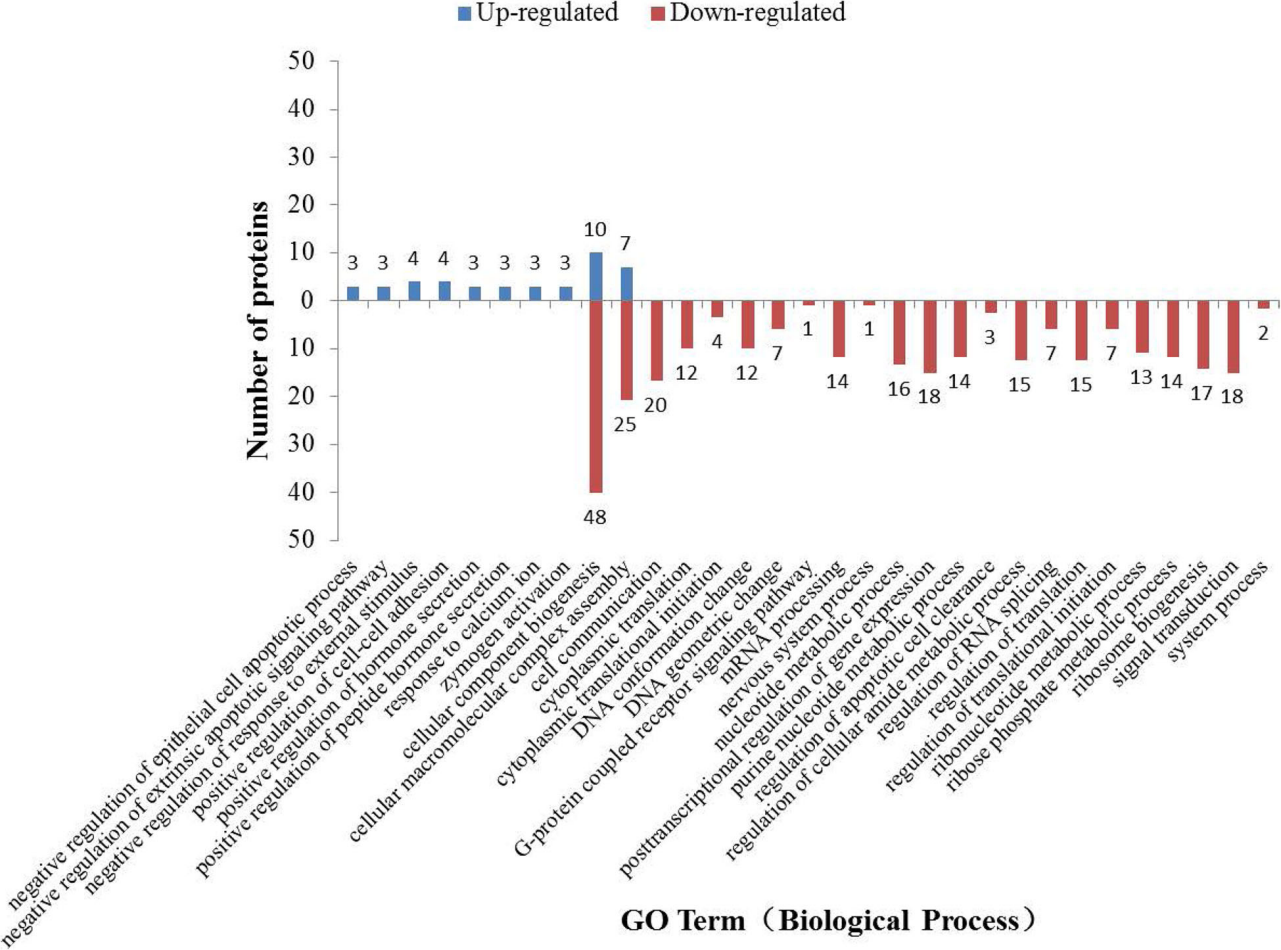
Biological processes of upregulated proteins and downregulated proteins

The differentially expressed proteins were categorized into 31 biological processes. Besides cellular component biogenesis and cellular macromolecular complex assembly, each of the other biochemical pathways includes either only up-regulated proteins or only down-regulated proteins (Fig. 2).

### KEGG pathway analysis

**Fig. 3:**
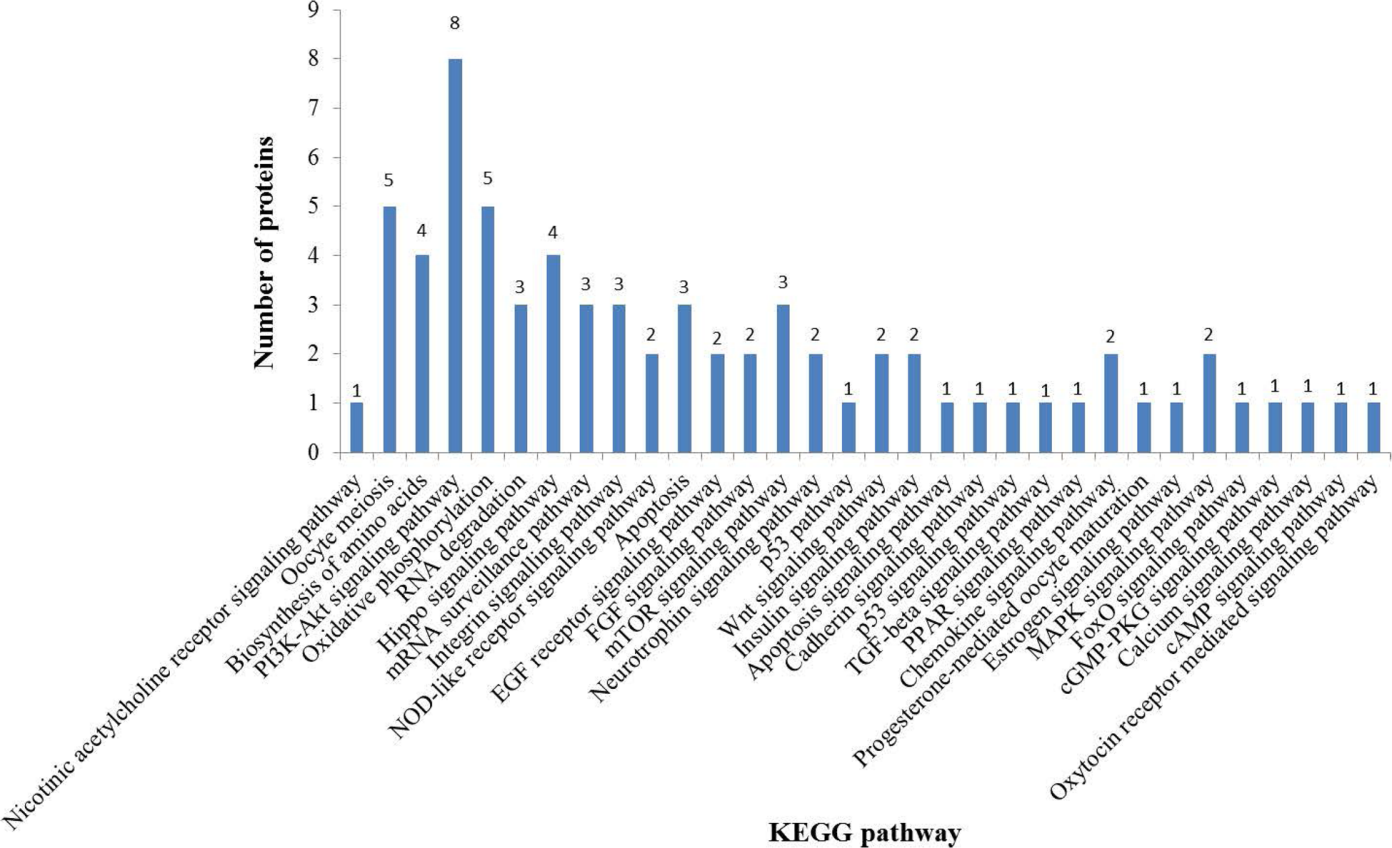
Differentially expressed proteins KEGG pathways analysis. The x-coordinate is the signaling pathway names, the y-coordinate is the number of proteins

KEGG pathway analysis showed that some important biochemical metabolic pathways and signal transduction pathways were associated with follicular development (Fig. 3), such as the Wnt signaling pathway, the estrogen signaling pathway, oocyte meiosis, progesterone-mediated oocyte maturation, the insulin signaling pathway, the FGF signaling pathway, the EGF receptor signaling pathway, apoptosis signaling pathway, the FoxO signaling pathway, and the p53 signaling pathway. Most importantly, the PI3K-Akt signaling pathway is a classical signaling pathway in follicular development regulation, including 8 differentially expressed proteins (HSP90AB1, GNG10, YWHAG, YWHAH, YWHAB, YWHAQ, RPS6, CDC37).

### Immunoblot validation

**Fig. 4:**
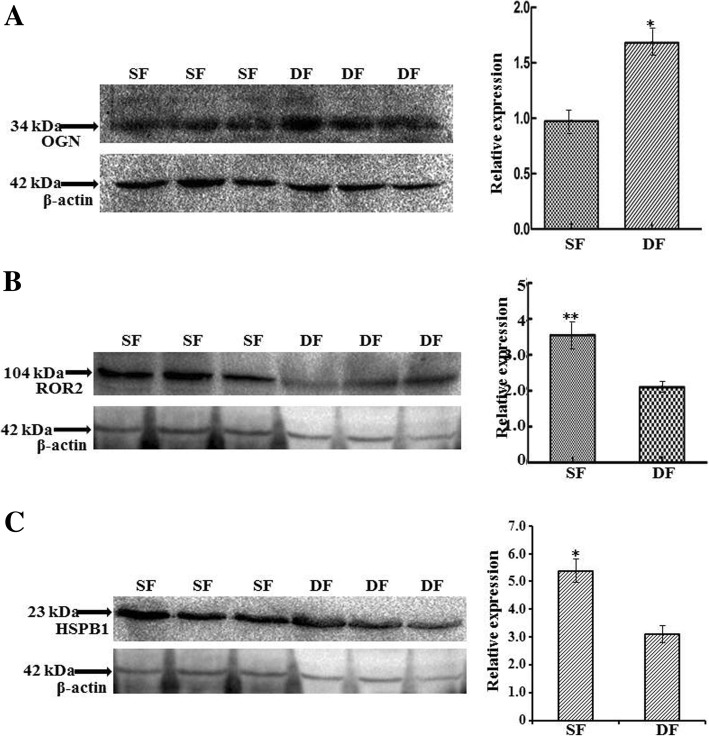
Western blots for validation of OGN, ROR2 and HSPB1 abundance in DF and SF. β-actin was used as a loading control, and the abundance of proteins was corrected relatively to β-actin. On the left side of **a**-**c**: immunoblot results of OGN, ROR2, HSPB1 and β-actin in DF and SF GCs; on the right side of **a**-**c**: protein relative expression level of OGN, ROR2 and HSPB1 in DF and SF GCs. Superscript single and double asterisk indicate significantly different at the 0.05 and 0.01 levels, respectively. (*n* = 3 each; least square mean ± SE)

Three randomly selected proteins including OGN, ROR2, and HSPB1 were analyzed via immunoblotting. OGN increased significantly (*P* < 0.05) in DF compared with SF, ROR2 increased extremely significantly (*P* < 0.01) in SF compared with DF, and HSPB1 increased significantly (*P* < 0.05) in SF compared with DF. The expression tendency of the three proteins were consistent with MS data (Fig. 4) (Additional file 2).

## Discussion

This study identified numerous new candidate proteins potentially interacting with the follicular development mechanism. A total of 3409 proteins were identified in both types of follicles and 259 of them were identified as differentially expressed between DF and SF.

Our study provided novel information in bovine follicles and identified specific highly differentially expressed proteins in GCs such as HSPA5, HSP90B1, HSPA8, HSPB1, HSPD1, ATP5B, ATP5A1 and EEF1A1. Heat shock protein (HSP) expression is fundamental in the reproductive system of mammals, and different HSP members were also found in ovaries [15]. Out of 40 highly expressed proteins, 5 HSPs (HSPA5, HSP90B1, HSPA8, HSPB1, HSPD1) were detected. As ATP5A1 and ATP5B are highly expressed in glioblastoma tumor cells [16], they may be closely related to cell proliferation. Eukaryotic translation elongation factor 1 alpha (EEF1A) is known to participate in protein synthesis, and one isoform of EEF1A is EEF1A1. It is possible that the 8 proteins mentioned above promote follicular growth by associating with cell proliferation.

Most differentially expressed proteins are down-regulated, consisting mainly of ribosomal proteins, eukaryotic translation initiation factors, and tubulin, and these proteins could promote cell growth and proliferation. SF and DF in earlier waves eventually undergo atresia, whereas they are down regulated only in DF. The question is uncertain but meaningful, so it needs to be further research.

GO analysis of biological processes of expressed proteins in DF and SF showed that numbers of expressed proteins associated with steroid biosynthetic process in DF were greater than in SF. It is known that GCs secrete steroid hormones, which are related to follicle maturation. It is suggested that more steroids may be needed for ovulation in DF than in SF. In addition, 28 proteins in DF were identified in the male gamete generation process, whereas no proteins in SF were detected in this biological process, so it is possible that DF begins the preparation for fertilization. Biological process classifications of differentially expressed proteins showed most of the upregulated proteins were involved in the negative regulation of the epithelial cell apoptotic process, negative regulation of the extrinsic apoptotic signaling pathway, positive regulation of the hormone secretion, and the positive regulation of peptide hormone secretion. Most of the downregulated proteins were, instead, related to cell communication, signal transduction, ribosome biogenesis, and regulation of translational initiation (EIF4A1, EIF5, EIF3I, EIF1). In addition, the FSH receptor [17, 18], estrogen receptor [19] and CART [20] receptor are all G protein-coupled receptors (GPCRs). We found that some proteins were involved in GPCR signaling pathways in both types of follicles, suggesting GPCRs play an essential role during follicular development. In addition, GTPases play vital functions in cellular trafficking, cell division, and translation [21], and many proteins involved in GTPase activity were found by the GO analysis results of molecular functions.

The eminent event in DF and SF during a bonine follicular wave is size difference, as DF continues to grow, while SF begins to regress. KEGG analysis revealed differentially expressed proteins (YWHAG, YWHAH, YWHAB, YWHAQ) involved in the Hippo signaling pathway that play an essential role in maintaining organizational size [22–24]. YWHAG, YWHAH, YWHAB, and YWHAQ belong to the 14–3-3 family of proteins, which are considered as anti-apoptotic and critical regulatory proteins in cell division and apoptosis. These proteins may also play an important role in regulating cellular activities by associating with cytoskeletal proteins [22]. It is clear that Hippo and Akt signaling pathways regulate follicle growth, but most ovarian follicles are restricted to growth under physiological conditions due to local Hippo signaling [25].

Oxidative stress may be related to folliculogenesis and oogenesis [26] in the bovine species. SOD2 – mitochondrial can act directly on superoxide anion radicals – for example, estradiol inhibited SOD2 mRNA expression in rat luteal cells [27]. KEGG analysis showed that SOD2 participate in the FoxO signaling pathway, and SOD2 was upregulated in DF. These results may indicate that SOD2 plays an important role in DF.

## Conclusions

The present study characterized the GCs proteome of bovine follicles at specific stages and screened 259 differentially expressed proteins in DF and SF. Many of these proteins may be linked to follicular development such as HSPA5, HSP90B1, HSPA8, HSPB1, HSPD1, ATP5B, ATP5A1, EEF1A1, 14–3-3 proteins (YWHAG, YWHAH, YWHAB, YWHAQ), and SOD2. Our findings will not only enrich the regulation theory of follicular development and dominance, but also have great significance to the application of propagation.

## Additional files

Additional file 1: 259 differentially expressed proteins. (XLSX 54 kb)

Additional file 2: Original immunoblotting results. (XLSX 9 kb)

## Data Availability

Availability of data and materials are included in the manuscript, figures, and tables.

## Abbreviations

AGC: Automatic gain control
BCA: Bradford Coomassie® Brilliant Blue G-250 method
BSA: Bovine serum albumin
CART: Cocaine and amphetamine-regulated transcript
CID: Collision-induced dissociation
DF: Dominant follicle
EEF1A: Eukaryotic translation elongation factor 1 alpha
FDR: False discovery rate
FSH: Follicle stimulating hormone
GCs: Granulosa cells
GO: Gene Ontology
GPCRs: G protein-coupled receptors
HSP: Heat shock protein
KEGG: Kyoto Encyclopedia of Genes and Genomes
LC-MS/MS: Capillary High Performance Liquid Chromatography coupled with label-free mass spectrometry
MS: Mass spectrometry
NC: Nitrocellulose
PANTHER: Protein Analysis Through Evolutionary Relationships
SDS-PAGE: Sodium dodecyl sulfate polyacrylamide gel electrophoresis
SF: Subordinate follicle
